# Stabilization of ferulic acid in topical gel formulation via nanoencapsulation and pH optimization

**DOI:** 10.1038/s41598-020-68732-6

**Published:** 2020-07-23

**Authors:** Surajit Das, Annie B. H. Wong

**Affiliations:** Institute of Chemical and Engineering Sciences, A*STAR (Agency for Science, Technology and Research), 1 Pesek Road, Jurong Island, 627833 Singapore

**Keywords:** Drug delivery, Colloids, Nanoparticles

## Abstract

Ferulic acid is a potent anti-oxidant with scientifically proven skin care efficacies. However, instability of this active in the skin care products restricted its wide application in beauty and skin care industries. This study aimed to stabilize ferulic acid in topical hydrogel formulation via nanoencapsulation technique. Ferulic acid loaded nanocapsules were prepared via high pressure homogenization method and physicochemically characterized. Mean particle size of ferulic acid loaded nanocapsules was < 300 nm. TEM and SEM images exhibited spherical particles with smooth surface. DSC and XRD results indicated that ferulic acid was completely dissolved in the lipid matrix of the nanocapsules and remained in amorphous form. Two types of hydrogel formulations containing ferulic acid loaded nanocapsules were prepared: Gel A with pH higher and Gel B with pH lower than p*Ka* of ferulic acid. Cross-polarized microscopic image of the gel formulations did not show presence of any un-encapsulated and un-dissolved crystal. Gel B showed slower and controlled release of ferulic acid than Gel A. Ferulic acid permeation through skin mimic from the gel formulation demonstrated controlled permeation. Color stability of the gel and chemical stability of ferulic acid were very good in Gel B, while poor in Gel A (although significantly better than the gel with un-encapsulated ferulic acid). The result clearly indicates that together with nanoencapsulation, low pH (less than p*Ka* of ferulic acid) of the hydrogel was crucial for both product appearance and chemical stability of ferulic acid. In fact, it has been proved that skin care product with low pH is good for skin as it can maintain skin homeostasis and microbiome. Furthermore, the permeation result suggests that ferulic acid may penetrate into deep skin layers and at the same time avoid systemic circulation. Overall, this low pH hydrogel formulation containing nanoencapsulated ferulic acid demonstrates great promise for commercialization.

## Introduction

Antioxidants are among the most abundant actives in skin care products, such as anti-aging, anti-pollution products. Antioxidants are used to prevent/delay skin aging caused by environmental aggressors like pollution, infrared (IR) radiation, and ultraviolet (UV) rays^1–4^. However, antioxidants are often associated with stability challenges when incorporated in the skin care products^3,5^. Antioxidants may partially or fully degrade into inactive forms during product shelf-life^6–8^. The degradation usually accelerates at high temperature (e.g., 40 °C) and under light (e.g., UV and fluorescent)^5,7,8^. This instability of the antioxidant actives ultimately reduces product’s efficacy and most of the time leads to product color change during storage^9,10^. Hence, consumer acceptance of the products also decreases.

In recent years, there is a huge interest on naturally occurring active ingredients for skin care products. One of the potent natural antioxidant is ferulic acid which is 4-hydroxy-3-methoxycinnamic acid^11^. Ferulic acid is a phytochemical which is omnipresent in nature (e.g., broccoli, spinach, carrot, orange, tomato, rice bran, and corn)^12^. It has strong free radical scavenging activity^13,14^. It has several skin care benefits^15^, such as anti-aging (including photo-aging)^3,16–18^, anti-wrinkle^19^, anti-pigmentation or skin lightening/whitening^19–21^, anti-collagenase^22,23^, anti-inflammatory^24^, antimicrobial^25^, and anti-tyrosinase^26^ efficacies. It is also able to absorb UV light and can provide photo-protection^17,27^. Therefore, ferulic acid can be used in anti-aging and sunscreen products^17^. However, its wide application in skin care and cosmetic products has been limited by its poor solubility and stability. Ferulic acid is poorly soluble in water (soluble in alkaline pH only)^17^. Moreover, ferulic acid has limited solubility in the oils/solvents which are suitable for skin care and cosmetic applications^17^, making it difficult to formulate in oil based formulations too. Ferulic acid readily degrades (primarily via decarboxylation) into 4-vinyl guaiacol (4-hydroxy-3-methoxystyrene) and other derivatives, such as 4-ethyl guaiacol, acetovanillone (4-Hydroxy-3-methoxyacetophenone), vanillin, vanillic acid and dimer of 4-hydroxy-3-methoxystyrene, which can change product color and reduce product efficacy^5,8,13^. It mainly degrades at high temperature, high relative humidity (RH > 76%), high pH, under UV light, and also in presence of several formulation excipients^5,7,8,28,29^. Although L’Oréal is using 0.5% (w/w) ferulic acid in their Skinceuticals (C E FERULIC and PHLORETIN CF)^9,10^ and Lancôme GÉNIFIQUE (Advanced Génifique Sensitive Serum)^30^ skin care brands, these products have some limitations. Skinceuticals products contain multiple antioxidant actives (e.g., ascorbic acid, vitamin E, phloretin) in addition to ferulic acid, which may help to improve stability of the actives. Surprisingly, these products also carry a special note indicating that the product may darken over time and may cause slight tingling sensation. This clearly suggests that degradation of the actives and/or color change of the products might still be substantial issues even if multiple antioxidants are combined in single product. Furthermore, most probably use of high amount of glycol (to dissolve the active) in the product might have caused the tingling sensation. Lancôme GÉNIFIQUE product (a dual antioxidant serum) also contains vitamin E (antioxidant) together with ferulic acid. In this product, ferulic acid solution is kept in a separate chamber that is isolated from the rest of the composition, which should be mixed before use. Furthermore, the label carries a special instruction (e.g., special storage condition (cold), short shelf life (two months) after mixing ferulic acid with rest of the formulation ingredients, etc.), most likely to avoid stability concerns of ferulic acid. Therefore, the stability challenge remains major concern for ferulic acid’s use in skin care and cosmetic products. This study focused on to solve this stability challenge in topical formulations.

One of the most realistic approaches to solve the stability issue of active is the encapsulation of the active^31–33^. Encapsulation of active may protect the active from the external factors (e.g., oxidation, hydrolysis, interaction with other formulation ingredients, direct exposure to light, etc.). However, size of the encapsulated particles is very important for topical products. It has been reported that particles > 10 µm remain on the skin surface, while particles between 3 and 10 µm deposited in the hair follicles and particles < 3 µm may penetrate via either hair follicles or stratum corneum^34,35^. Nevertheless, the encapsulated particles in skin care products should be sufficiently small so that the capsules cannot be felt (negative sensory) during product application on skin. Previously, researchers encapsulated ferulic acid in polymeric nanoparticle and prepared hydrogel^36^. However, the focus of that study was to find the formulation’s efficacy for wound healing. The study did not target to stabilize ferulic acid in the gel formulation and find its usefulness for skin-care/cosmetics application. Recently, we investigated formulation of ferulic acid co-crystal in oleogel formation for skin care application^37^. However, most of the skin care products are either aqueous based or O/W emulsion or O/W emulsion based cream. Therefore, stabilization of ferulic acid in water-based topical formulation (e.g., hydrogel) will be impactful to the beauty and skin care industries.

Aim of this study is to improve ferulic acid’s stability in topical hydrogel formulations by nanoencapsulating ferulic acid before its addition to the rest of the formulation. Encapsulation of ferulic acid is expected to improve its physicochemical stability in the formulations by protecting it from external aggressors during storage. In this study, ferulic acid was intended to be encapsulated in the lipid excipient which has several advantages over polymeric nanoparticles^38^. First, appropriate lipid excipient was selected based on the solubility of ferulic acid in the lipid excipients. Then nanoencapsulated ferulic acid was prepared by high pressure homogenization method following optimized formulation and process variables. Nanoencapsulated ferulic acid was physicochemically characterized via particle size and polydispersity index (PdI) analysis, cross-polarized light microscopy, differential scanning calorimetry (DSC), X-ray diffraction spectroscopy (XRD), transmission electron microscopy (TEM), scanning electron microscopy (SEM) and high performance liquid chromatography (HPLC). Two types of hydrogel formulations of the nanoencapsulated ferulic acid were prepared: one at pH lower and another at pH higher than p*Ka* of ferulic acid. Franz diffusion cell studies were conducted to evaluate in vitro release and permeation of ferulic acid from the hydrogel formulations. Finally, physicochemical stability of the formulations was investigated at different storage conditions.

## Materials and methods

### Materials

Ferulic acid, citric acid, 2-amino-2-methyl-1-propanol (AMP), sodium hydroxide (NaOH), potassium chloride (KCl), potassium dihydrogen phosphate 98% (KH_2_PO_4_), sodium phosphate dibasic ≥ 99% (Na_2_HPO_4_), and isopropyl myristate were purchased from Sigma (USA). Compritol 888 ATO, Monosteol, Precirol ATO5, Geleol FPF, Geleol Mono and Diglyceride, Geleol Pastilles, Compritol HD5 ATO, Sedefos 75, Gelot 64, Tefose 63, Gelucire 44/14, and Gelucire 50/13 were gift samples from Gattefossé (France). Myristyl alcohol was obtained from Kokyu Alcohol Kogyo Co. Ltd (Japan). HPLC grade acetonitrile and methanol were obtained from Duksan (South Korea). Tetrahydrofuran was purchased from VWR (USA). Acetic acid, sodium chloride (NaCl), cetyl alcohol, and Strat-M membrane were bought from Merck (USA). Carbopol Aqua CC polymer and Carbopol Ultrez 21 polymer were received from Lubrizol (USA). Ethomeen O/12LC was purchased from Akzo Nobel (Netherlands). Xanthan gum was obtained from CP Kelco (USA). Mixture of phenoxyethanol and ethylhexylglycerin was received from Schülke & Mayr GmbH (Germany). Water used in this study was ultrapure water from Milli-Q Gradient A10 system (Millipore, France).

### Measurement of ferulic acid solubility in the lipid excipients

Solubility of ferulic acid in the solid lipid excipients were checked via an established method^31^. Briefly, 50 mg ferulic acid and ~ 200 mg of lipid excipient were accurately weighed in a screw capped clear glass vial. The vial was then heated at 80 °C to melt the lipid excipient and continuously agitated on magnetic stirrer to dissolve ferulic acid in the molten lipid excipient. Then additional lipid excipient was added part by part with continuous agitation at 80 °C until the suspension turns into a clear solution. The amount of lipid excipient added to obtain this clear solution was noted down. Compritol 888 ATO, Monosteol, Precirol ATO5, Geleol FPF, Geleol Mono and Diglyceride, Geleol Pastilles, Compritol HD5 ATO, cetyl alcohol, myristyl alcohol, Sedefos 75, Gelot 64, Tefose 63, Gelucire 44/14 and Gelucire 50/13 were tested as lipid excipients.

### Nanoencapsulation

Nanoencapsulation of ferulic acid was performed by high pressure homogenization method^38^. Briefly, lipid excipient was weighed in a beaker and melted at 70 °C (> 5 °C above the melting point of the lipid excipient). Accurately weighed ferulic acid was added in the melted lipid excipient and mixed well to completely dissolve. In a separate beaker, accurately weighed ultrapure water was heated at 70 °C (beaker was covered with aluminum foil to prevent evaporation of water). Hot water was slowly added in the melted lipid containing ferulic acid and homogeneously dispersed by IKA T-10 basic Ultra-Turrax disperser (Germany) at ~ 14,000–15,000 rpm for 5 min at 70 °C. Then the dispersion was homogenized by Avestin EmulsiFlex C5 high-pressure homogenizer (Canada) at 800 bar. The homogenizer was placed in a water bath at 70 °C to maintain the temperature throughout the process. The homogenization process was repeated for 5 cycles. The hot nanodispersion was collected and placed on an ice bath to cool down rapidly. In a recent study, we investigated this nanoencapsulated system at molecular-level, which showed successful encapsulation of ferulic acid in the nanocapsules composed of Gelucire 50/13^39^.

### Particle size and size distribution measurements

Particle size (z-average diameter) and polydispersity index (PdI) were measured at 25 °C by Malvern Zetasizer Nano ZS (Malvern Instruments, UK) following dynamic light scattering principle^40^. The instrument carries a 4 mW He–Ne laser that operates at 633 nm wavelength. The measurement was conducted at 173° detection angle. The nanodispersions were measured without and with 10-times dilution with water.

### Differential scanning calorimetry analysis

Lyophilization of nanoencapsulated active (ferulic acid) dispersion and empty nanocapsule dispersion (ferulic acid free) was performed in Vir Tis benchtop lyophilizer (USA). The nanoencapsulated ferulic acid and the empty nanocapsule were lyophilized to protect their physical state. Differential scanning calorimetry (DSC) analyses were carried out in TA Instruments SDT-Q600 Simultaneous TGA/DSC (USA). The thermograms of ferulic acid, lipid excipient (Gelucire 50/13) and lyophilized nanocapsules were recorded. Briefly, samples (~ 4 to 5 mg) were sealed in standard aluminum pans and kept under isothermal condition at 25 °C for 15 min. Then the temperature was increased from 25 to 200 °C at 10 °C min^−1^ under a nitrogen atmosphere. An empty sealed pan was used as reference.

### X-ray diffraction study

Similar to DSC, lyophilization of nanoencapsulated active (ferulic acid) dispersion and empty nanocapsule dispersion (ferulic acid free) was performed in VirTis benchtop lyophilizer (USA). Powder X-ray diffraction (PXRD) measurements were carried out by Bruker D8-ADVANCE powder X-ray diffractometer (Germany) using Cu K*α* radiation as X-ray source. The X-ray diffractograms of ferulic acid, lipid excipient (Gelucire 50/13) and lyophilized nanocapsules were recorded. Briefly, samples were first kept in the glass sample holders and then scanned from 2° to 80° with 2° min^−1^ scan angular speed (2*θ* min^−1^) at 0.02° step size. Operating voltage and current were set as 35 kV and 40 mA, respectively.

### Cryogenic transmission electron microscopy

Cryogenic transmission electron microscopy (cryo-TEM) was used to evaluate the shape and morphological features of the nanocapsules. Just before sample preparation, the grid (Quantifoil, R2/2, Holey carbon film) was glow-discharged at 20 mA for 60 s to increase its hydrophilicity. The sample was prepared in a vitrification robot (FEI Vitrobot Mark IV, USA). Briefly, 5 µL nanocapsule dispersion was applied onto the grid and excess sample was blotted away with filter paper (blotting time of 2 s) at room temperature under 100% humidity. This process led to a thin film formation on the grid. The sample was then plunged and vitrified in liquid ethane condensed by liquid nitrogen. The cryo-TEM study of the sample was conducted using FEI Titan Krios (USA) equipped with a field emission gun operating at 300 kV and the images were captured through Falcon II camera (4 k × 4 k).

### Scanning electron microscopy

Field Emission Scanning Electron Microscopy (FESEM) study was conducted on ferulic acid powder and Gelucire 50/13 (crushed). Briefly, sample was placed on a copper stub using a double-sided adhesive tape. Then the sample was sputter-coated with gold for 120 s at 20 mA and analyzed in FESEM (JEOL JSM-6700F, Japan) at 5 kV excitation voltage. Cryogenic FESEM (cryo-FESEM) technique was used to investigate the morphology of the nanocapsules. Cryo-FESEM is expected to analyze the liquid samples close to their original state^32^. For this, two–three drops of the nanosuspension were placed on a copper rivet and inserted in the liquid nitrogen chamber at −196 °C to freeze the sample. The frozen sample was quickly shifted into the cryo preparation chamber (Alto CT2500, Gatan, UK) under vacuum and freeze fractured with a knife at −95 °C on a cryo stage. The sample was sputter coated with platinum for 180 s and transferred into the specimen stage of the cryo-FESEM (JEOL JSM-6700F, Japan) at −140 °C. The sample was then analyzed at an excitation voltage of 5 kV.

### High performance liquid chromatography (HPLC) assay

Agilent HPLC (Agilent 1,100 series; USA) was used in this study. A reverse phase C18 column (ZORBAX Eclipse Plus C18; 5 µm; 250 mm × 4.6 mm; Agilent, USA) was used^37^. Temperature of the column was set at 25 °C. Tetrahydrofuran (THF) : Acetic acid (CH_3_COOH) : ultrapure water at 13:11:76 ratio was used as mobile phase at 1.5 mL min^−1^ flow rate (isocratic). Injection volume of the samples was 10 µL and detection wavelength was set at 318 nm. Ferulic acid peak appeared at ~ 6.2 min. The calibration curve was liner (*r*^*2*^ = 0.9998) at 1–100 µg mL^−1^ ferulic acid concentration range.

### Gel formulations of nanoencapsulated ferulic acid

Two types of gel formulations containing nanoencapsulated ferulic acid were prepared: Gel A and Gel B. Before preparing the gel formulations, nanoencapsulated ferulic acid dispersion was prepared as mentioned in earlier section. Composition of the nanoencapsulated ferulic acid dispersion was: 11.70% (w/w) Gelucire 50/13 as lipid excipient, 0.58% (w/w) ferulic acid and 87.72% (w/w) ultrapure water. This composition was selected so that final gel formulations (Gel A and Gel B) contain 10% (w/w) Gelucire 50/13 and 0.5% (w/w) ferulic acid.

*Gel A*: 0.4% (w/w) Carbopol Ultrez 21 polymer (Acrylates/C10-30 Alkyl Acrylate Crosspolymer) was added in 13.35% (w/w) ultrapure water and kept for 30 min to allow swelling of Carbopol polymer. Then 85.5% (w/w) nanoencapsulated ferulic acid dispersion was slowly added to the polymer dispersion and mixed using IKA overhead mixer (Germany) at 100 rpm until homogeneously mixed. After that 0.25% AMP (2-amino-2-methyl-1-propanol) was added drop wise while mixing. Then 0.5% (w/w) preservative (mixture Phenoxyethanol and Ethylhexylglycerin) was added and continued mixing for another 30 min.

*Gel B*: 10% (w/w) Carbopol Aqua CC polymer (Polyacrylate-1 Crosspolymer) was slowly added in 85.5% (w/w) nanodispersion and mixed using IKA overhead mixer (Germany) at 100 rpm until homogeneously mixed. Then 4% (w/w) citric acid stock solution (25% (w/w) citric acid in ultrapure water) was slowly added while mixing. After that 0.5% (w/w) preservative (mixture Phenoxyethanol and Ethylhexylglycerin) was added and continued mixing for another 30 min. Carbopol Aqua CC polymer is in liquid form, which contains 20% active polymer.

*Gel C*: Gel C is exactly same as Gel B except % ferulic acid. Composition of the nanoencapsulated ferulic acid dispersion used for Gel C was: 11.70% (w/w) Gelucire 50/13 as lipid excipient, 0.29% (w/w) ferulic acid and 88.01% (w/w) ultrapure water. Therefore, Gel C contains 0.25% (w/w) ferulic acid instead of 0.5% (w/w). However, percentage of Gelucire 50/13 was same as Gel B (i.e., 10% (w/w)).

The viscosity of the gel formulations were measured by a viscometer (Brookfield DV2T; Canada) at 10 rpm using SC4-15 spindle at room temperature (23–25 °C).

### Ferulic acid aqueous solution and its gel formulation

As ferulic acid has poor aqueous solubility^41^, 0.5% (w/w) ferulic acid aqueous solution was prepared via neutralization method (ferulic acid is soluble in water at alkaline pH). Briefly, 0.5 g ferulic acid was dispersed in 80 g ultrapure water in a beaker. Sodium hydroxide aqueous solution (1% w/w) was added drop wise while stirring on magnetic stirrer until ferulic acid was completely dissolved. Then required amount of ultrapure water was added to adjust the solution to 100 g to make it 0.5% (w/w) ferulic acid solution. For ferulic acid aqueous gel formulation, 0.8 g water was replaced with 0.8 g xanthan gum in the above ferulic acid solution. Xanthan gum was slowly added and mixed well. These formulations were prepared as reference samples for stability study. Ferulic acid aqueous solution and its gel formulation cannot be prepared at low pH as ferulic acid precipitates out at acidic pH due to poor solubility.

### Cross-polarized light microscopy

Aqueous dispersion of nanoencapsulated ferulic acid and its gel formulations were examined under cross-polarized light microscope (Olympus, Japan) to investigate the presence of any un-encapsulated ferulic acid crystal^31^. Briefly, small amount of the sample was placed on a glass slide and covered with a cover slip to form a thin uniform film on the glass slide. The slide was then put on the microscope stage and the sample was evaluated under cross-polarized light. The images were captured by the attached digital camera (Nikkon, Japan) and processed by NIS-Elements AR 4.60.00 software. In addition to the freshly prepared samples, the gel formulations stored at 5 °C for three months were also investigated under cross polarized microscope. The samples stored at 5 °C were selected as the chance of crystallization of ferulic acid is high at low temperature (less solubility at low temperature). On the other hand, ferulic acid powder was directly mixed in Carbopol Aqua CC gel (same composition as Gel B except ferulic acid) and investigated under cross-polarized light microscope as reference sample.

### In vitro release and skin permeation studies

In vitro release and skin permeation studies were performed using Franz diffusion cell (Teledyne Hanson Research, 6-cell manual diffusion test system, standard 7 ml vertical diffusion cell; USA). Briefly, Franz diffusion cells were placed on a multistage magnetic stirrer. The cells were connected with water circulator where the water temperature was set at 37.5 °C. Mixture of Phosphate buffered saline (PBS; pH 7.4) and methanol at 80:20 volume ratio was used as receptor fluid to maintain sink condition. Hydro-alcoholic solution is commonly used for release/permeation study of poorly soluble drug to maintain sink condition^37,42^. HT Tuffryn Polysulfone membrane with 0.45 µm pore size and 25 mm diameter (Pall Corporation, USA) was used for release test^42^ and Strat-M was used for skin permeation study^43^. The membranes were placed in between donor chamber and receptor chamber, which isolates the gel formulation from receptor fluid. Gel formulation was placed in the donor chamber and receptor fluid was placed in the receptor chambers. Magnetic stirrer speed was set at 300 rpm. Water circulator was switched on to maintain receptor fluid’s temperature at 37.0 ± 0.5 °C. Samples (receptor fluid) were withdrawn from the sampling port at 0.5, 1, 2, 3, 4, 5, 6, and 24 h for HPLC analysis. For the sample withdrawal, 2 mL fresh receptor fluid was injected through the bottom port after switching off the magnetic stirrer. First 1 mL sample was discarded and then the sample was collected in the HPLC vial from the top sampling port. Magnetic stirrer was switched on after sample collection. After 24 h sample collection, the membranes were taken out from the Franz diffusion cells and investigated under microscope. Membranes showed no damage and were intact.

### Stability

As mentioned in introduction, ferulic acid has immense stability concern in topical products (especially in aqueous based liquid/semisolid products), which may also change product color^8,13,28^. Hence, investigation of both physical and chemical stability of the formulations at different stability conditions is crucial as products are required to be stable during their shelf-life. Nanoencapsulated ferulic acid dispersion and nanoencapsulated ferulic acid containing gel formulations were stored at 5 ± 3 °C, 25 °C, 40 °C/75% RH (relative humidity), 50 °C, and under UV light (combination of 254 nm and 365 nm wavelength). Stability of the formulations were evaluated on first day (i.e., freshly prepared formulation), after two weeks (only product colors were evaluated), one month, and three months (nanoencapsulated ferulic acid dispersion as well as gel samples stored at 50 °C were not evaluated after three months). Stability was investigated as follows: i) product colors were checked visually, ii) pH of the products stored at 40 °C/75% RH were measured by pH meter (Mettler Toledo, USA), and iii) active contents of all samples were analyzed by HPLC. For active content analysis, the formulations were accurately weighed and dissolved in required amount of methanol in the volumetric flask. The mixture was then sonicated for 30 min for complete extraction of ferulic acid in methanol. Then the methanolic solution was filtered through 0.45 µ PTFE syringe filter (Thermo Scientific, USA) and analyzed by HPLC as mentioned earlier. Ferulic acid aqueous solution and ferulic acid aqueous gel were also stored at the similar storage conditions to monitor stability after two weeks and one month.

### Statistical analysis

All statistical analyses were performed in Microscoft Excel 2016. Experimental data were expressed as mean ± standard deviation (SD). The results were analyzed following t-test: paired two samples of means analysis (two-tail). Statistical significance was set at *p* < 0.05.

## Results and discussion

### Solubility of active in the lipid excipients

**Figure 1:**
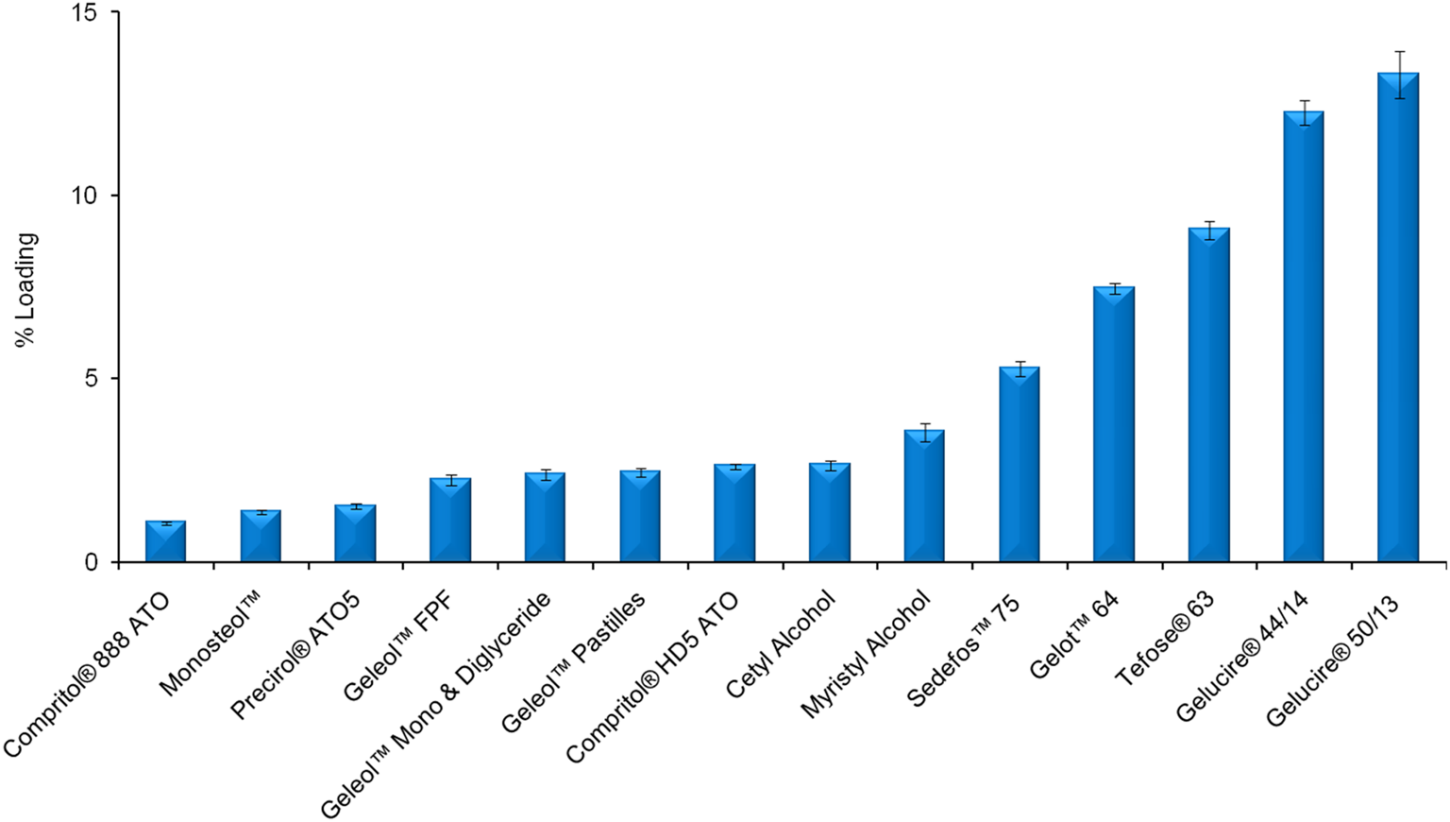
Ferulic acid solubility in the lipid excipients (% Loading represents weight % of dissolved ferulic acid in compare to the amount of lipid excipients). Data represent mean ± SD (n = 3).

Determination of ferulic acid solubility in the lipid excipients is the important first step to identify the suitable lipid excipient for active encapsulation. In general, active encapsulation efficiency or loading is proportional to the solubility of the active in the lipid excipients. Solubility of ferulic acid in the lipid excipients should be high enough to incorporate high amount of ferulic acid in the final formulation. Several commercial skin care products contain 0.5% ferulic acid (e.g., Skinceuticals C E FERULIC and Skinceuticals PHLORETIN CF)^9,10^. Therefore, this work aimed to select lipid excipients which can encapsulate enough amount of ferulic acid so that final topical formulation contains at least 0.5% ferulic acid. Several lipid excipients (as mentioned in Fig. 1) were tested for this purpose. Gelucire 50/13 (Stearoyl polyoxyl-32 glycerides) exhibited highest solubilization capability among the tested lipid excipients. Therefore, Gelucire 50/13 was chosen for the nanoencapsulation of ferulic acid.

### Particle size and size distribution

Particle size (z average diameter) and polydispersity index (PdI) of active-free nanodispersion and nanoencapsulated ferulic acid dispersion were compared (Supplementary Figure S1). Both nanodispersions contain 11.7% (w/w) Gelucire 50/13 as lipid excipient. 0.58% (w/w) ferulic acid was encapsulated in the nanoencapsulated ferulic acid dispersion. Particle size significantly increased after ferulic acid encapsulation while PdI decreased. Particle size was 57.7 ± 0.2 nm and PdI was 0.623 ± 0.006 for active-free nanodispersion when nanodispersions were measured without dilution (Supplementary Figure S1a). Particle size significantly increased to 291.2 ± 5.4 nm (*p* = 0.0002), while PdI directionally decreased to 0.380 ± 0.100 (*p* = 0.0504) in case of nanoencapsulated ferulic acid dispersion. Therefore, encapsulation of active increased particle size. High PdI was mainly due to multimodal distribution of particle size. One small peak at < 20 nm size was observed in the nanocapsule dispersions, which indicates the presence of micelles in addition to nanocapsules. In contrary to other solid lipid nanoparticles where both lipid(s) and surfactant(s) are required to produce the nanoparticles^31–33^, these nanocapsules were prepared with only single lipid excipient without any surfactant. This was possible as Gelucire 50/13 itself has surfactant property. Therefore, the presence of micelles together with the nanocapsules is not surprising. When 10-times diluted samples were measured, particle size and PdI were 38.3 ± 0.9 nm and 0.429 ± 0.013 for the active-free nanodispersion, respectively (Supplementary Figure S1b). Particle size and PdI significantly increased to 72.5 ± 0.8 nm (*p* = 0.0002) and 0.563 ± 0.005 (*p* = 0.0047) in case of the nanoencapsulated ferulic acid dispersion. The dilution of the nanodispersions with water (common practice) before size and PdI measurements influenced the distribution of micelle and nanocapsule in the dispersion, which led to different results between undiluted and diluted samples.

### Differential scanning calorimetry

**Figure 2:**
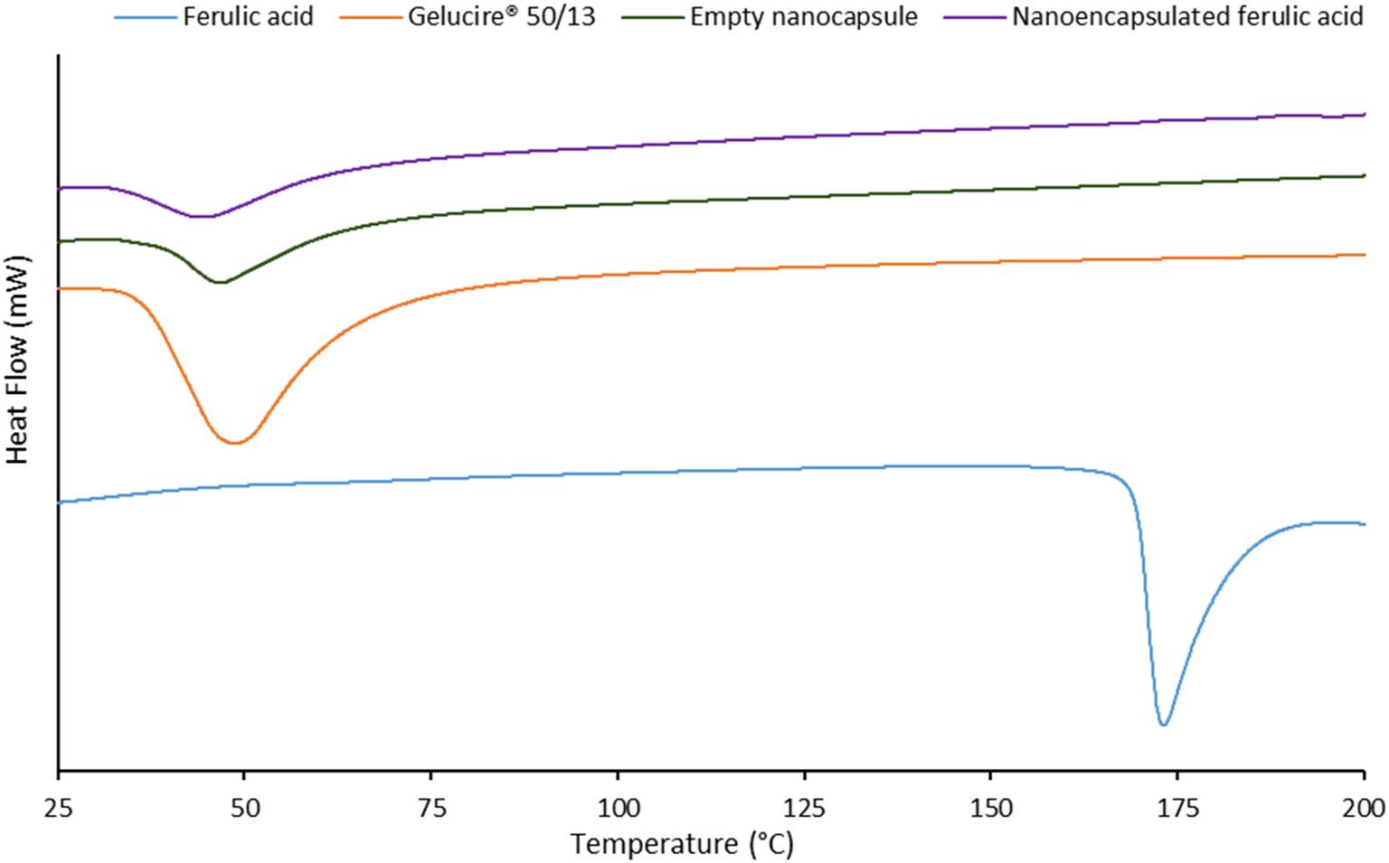
Thermogram of ferulic acid (active), Gelucire 50/13 (lipid excipient), empty nanocapsule (ferulic acid free), and nanoencapsulated ferulic acid.

Figure 2 represents the thermograms of ferulic acid, Gelucire 50/13 (lipid excipient), empty nanocapsule and nanoencapsulated ferulic acid. Crystalline ferulic acid showed a sharp endothermic peak at 173 °C (melting temperature of ferulic acid). Gelucire 50/13 showed a broad endothermic peak at 48.7 °C, while empty nanocapsule showed a broad endothermic peak at 46.8 °C. This slight shift in peak versus Gelucire 50/13 was probably due to nanoparticle formation where the lipid structure might have been slightly modified. Nanoencapsulated ferulic acid exhibited a broad endothermic peak at 44.1 °C. This minor shift of peak versus empty nanocapsule might be due to active loading in the lipid matrix, which probably caused slight modification of the lipid matrix. Furthermore, the peak height and area of both nanocapsules decreased in compare to pure lipid excipient. This should be due to reduced crystallinity of the lipid excipient upon nanoparticle formation. Similar observations were also noticed in previous works^32,33^. Absence of the peak at 173 °C in nanoencapsulated ferulic acid indicates one of the following two scenarios: i) ferulic acid crystals transformed to amorphous form in the lipid matrix or ii) ferulic acid completely dissolved in the lipid matrix upon heating. This can be clarified by PXRD result.

### X-ray diffraction

**Figure 3:**
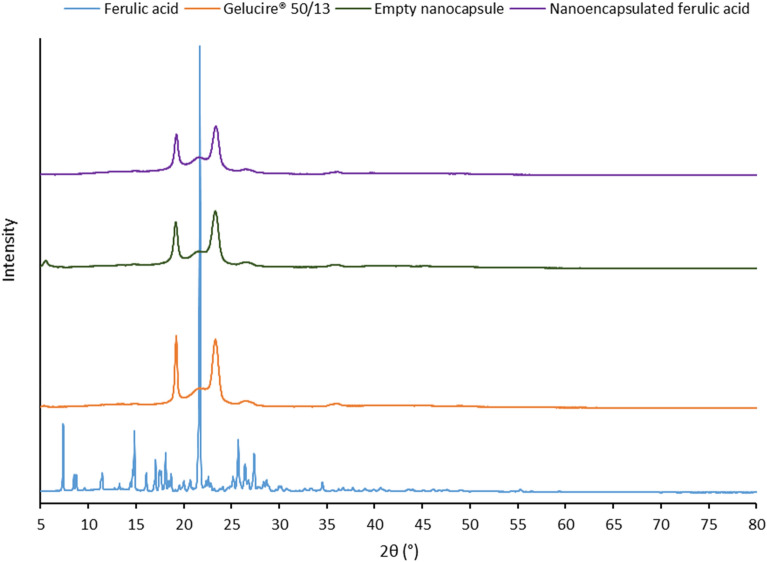
PXRD profile of ferulic acid (active), Gelucire 50/13 (lipid excipient), empty nanocapsule (ferulic acid free), and nanoencapsulated ferulic acid.

PXRD data (Fig. 3) confirmed the results demonstrated by DSC study. The diffraction pattern of ferulic acid showed a large crystalline peak at 2θ = 21.7°, few distinct sharp crystalline peaks at 7.4°, 14.8°, 17.0°, 18.1°, 25.7°, 27.4°, and few other low intensity peaks between 7.4° and 34.6°. These peaks were not detected in the diffractogram of the nanoencapsulated ferulic acid. This indicates that ferulic acid was completely dissolved in the lipid matrix of the nanocapsules and remained in amorphous form. Due to poor aqueous solubility^41^, crystalline peaks of ferulic acid would have been observed if ferulic acid located outside the lipid matrix. This suggests that the ferulic acid was successfully encapsulated. Two sharp peaks at 19.2° and 23.3° were observed for Gelucire 50/13. Diffraction pattern of the nanocapsules was similar to Gelucire 50/13 but intensity of the peaks was lower in the nanocapsules. This indicates lower crystallinity of the lipid matrix due to less ordered structure in the nanocapsules in compare to lipid excipient alone. This supports the observation from DSC study. Diffraction patterns of empty nanocapsule and nanoencapsulated ferulic acid are not significantly different, which indicates that encapsulation of ferulic acid did not significantly change the lipid matrix structure of the nanocapsules.

### Cryogenic transmission electron microscopy

**Figure 4:**
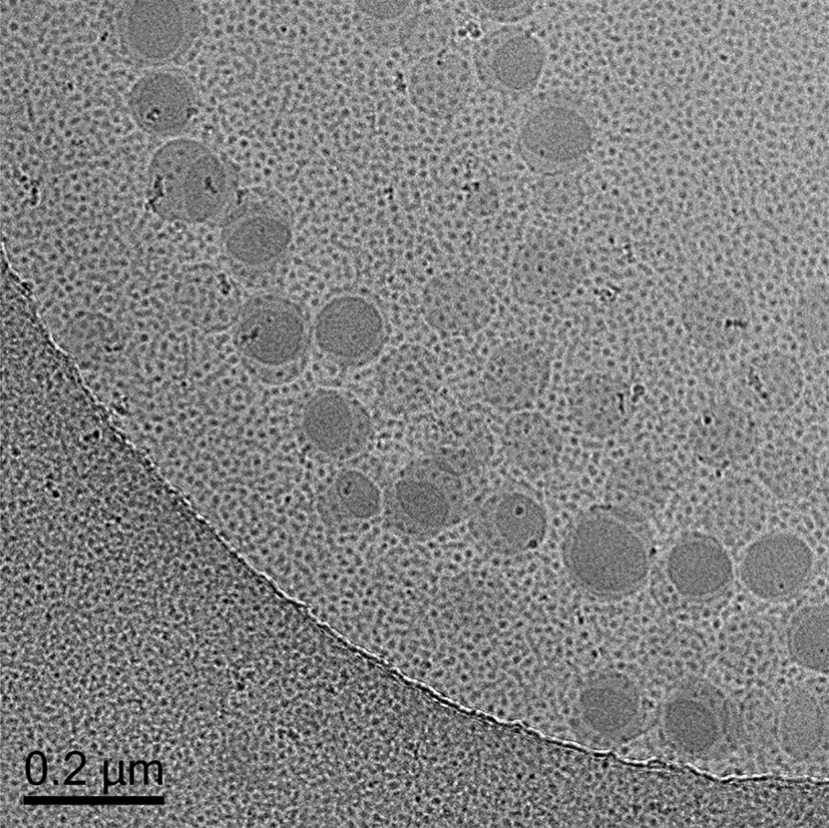
Cryo-TEM image of nanoencapsulated ferulic acid (×29,000 magnification).

To investigate the samples in their natural state cryo-TEM was utilized instead of TEM^44,45^. Figure 4 shows spherical particles. It also shows that the particles are well dispersed in the system and do not form agglomerates.

### Scanning electron microscopy

**Figure 5:**
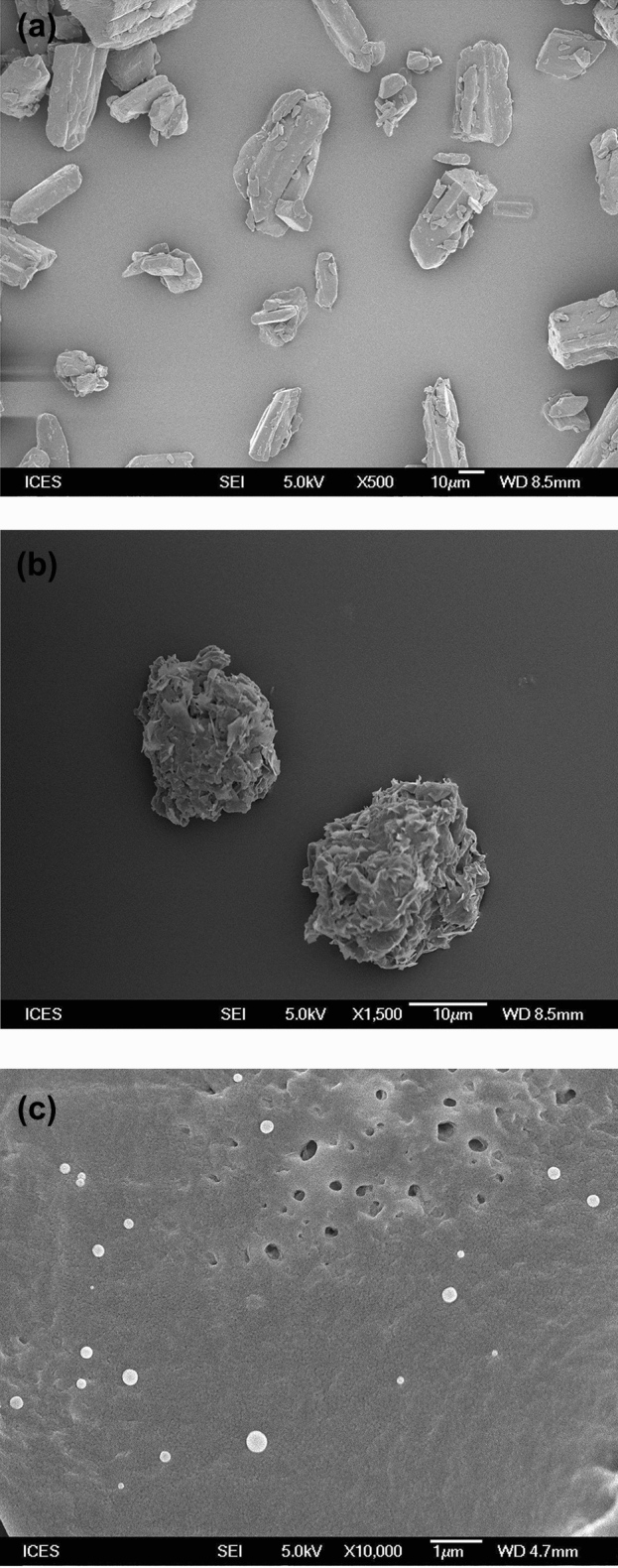
FESEM image of (**a**) ferulic acid powder, (**b**) Gelucire 50/13, and (**c**) cryo-FESEM image of nanoencapsulated ferulic acid dispersion.

FESEM images reveal crystalline shape of ferulic acid (Fig. 5a) and irregular shape of Gelucire 50/13 (Fig. 5b). Cryo-FESEM image of the nanoencapsulated ferulic acid dispersion exhibits spherical shape and smooth surface morphology of the nanocapsules (Fig. 5c). Crystalline structure of ferulic acid is absent in nanocapsule dispersion, which suggests the absence of un-encapsulated ferulic acid in the dispersion.

### Gel formulations

For topical application, semisolid formulation is preferred versus liquid formulation due to several advantages^46^. Therefore, hydrogel formulations were prepared for this study. The p*K*_a_ value of ferulic acid is 4.61^41^. Two types of gel formulations containing nanoencapsulated ferulic acid were prepared: i) one at pH above p*K*_a_ of ferulic acid (Gel A) and ii) another at pH below p*K*_a_ of ferulic acid (Gel B). Appearance of freshly prepared two gel formulations (Gel A and Gel B) was translucent to opaque. The pH of freshly prepared Gel A was 5.43 ± 0.11 and Gel B was 3.41 ± 0.07. It has been scientifically proved that pH of healthy skin surface is slightly acidic with pH < 5^47^. It is desirable to maintain this acidic pH to preserve skin homeostasis and microbiome. When this acid mantle is disrupted, it helps to grow harmful bacteria and also leads to adverse skin conditions (e.g., eczema, acne, dermatitis, etc.)^48,49^. Therefore, skin care and cosmetic products with low pH (pH < 5) is advantageous as it can help to maintain acidic pH of healthy skin. Hence, the developed Gel B has additional advantages for skin care and cosmetic application. The viscosities of Gel A and Gel B were 35,972 ± 639 and 17,843 ± 857, respectively. As mentioned earlier, several commercial skin care products contain 0.5% ferulic acid (e.g., Skinceuticals C E FERULIC, Skinceuticals PHLORETIN CF)^9,10^. Both Gel A and Gel B also contain 0.5% (w/w) ferulic acid. Another nanoencapsulated ferulic acid loaded gel formulation (Gel C) containing 0.25% (w/w) ferulic acid and 10% (w/w) Gelucire 50/13 at low pH was also prepared to find out the effect of % active loading in the nanocapsule on membrane permeation.

### Microscopic analysis

Cross polarized microscopy is an established technique to detect the presence of any crystal in the formulation^31^. First, aqueous dispersion of nanoencapsulated ferulic acid (contain 0.58% (w/w) ferulic acid and 11.7% (w/w) Gelucire 50/13) was checked under the cross-polarized microscope and absence of un-encapsulated ferulic acid crystals was confirmed. Then Gel A and Gel B containing nanoencapsulated ferulic acid (contain 0.5% (w/w) ferulic acid) were investigated and no crystal was observed. Furthermore, Gel A and Gel B containing nanoencapsulated ferulic acid stored at 5 °C for three months were also investigated under the cross-polarized microscope. Crystals were not observed in both gels (Supplementary Figure S2a represents Gel B). On the other hand, ferulic acid crystals were visible under cross polarized microscope when ferulic acid powder was simply mixed with Carbopol Aqua CC gel (Supplementary Figure S2b).This observation indicates that ferulic acid did not leak out and re-crystallize in the gel formulation even when stored at 5 °C for three months. This also eliminates the chance of negative sensory due to the presence of ferulic acid crystals (e.g., gritty feel on skin during application).

### Active release

**Figure 6:**
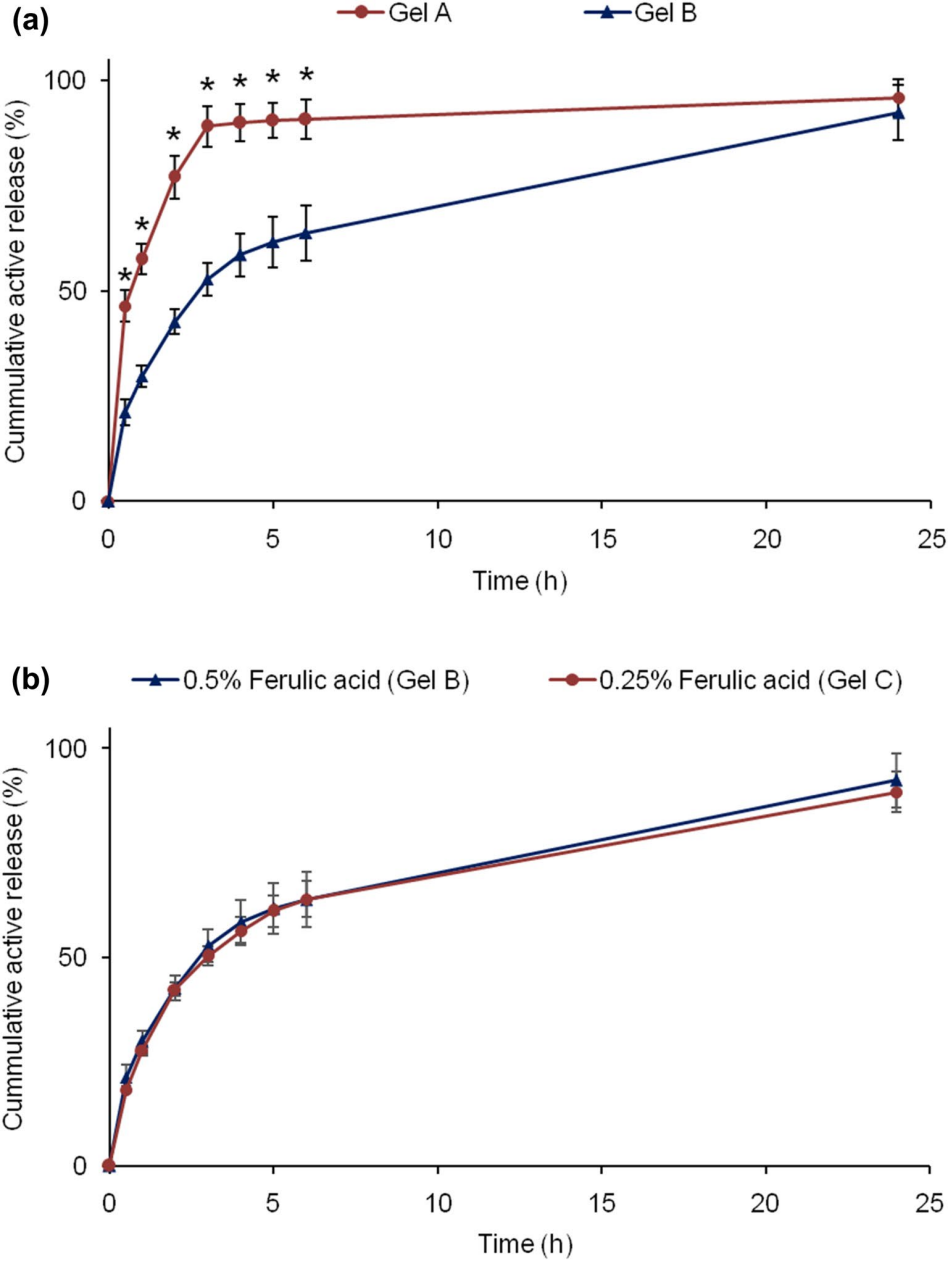
Cumulative active release (%) from the gel formulations containing nanoencapsulated ferulic acid: (**a**) Gel A versus Gel B and (**b**) 0.5% (Gel B) versus 0.25% (Gel C) nanoencapsulated ferulic acid gel. Data represent mean ± SD (n = 3). *Significant difference: Gel A versus Gel B (*p* < 0.05).

This study is critical as the active needs to be released from the formulation and penetrate through stratum corneum so that the active can act at the site of action (inner epidermis and dermis). Here polysulfone membrane with 0.45 µm pore size was used as barrier between hydrogel formulation and release media. Release of ferulic acid from gel formulation followed by permeation through the membrane to receptor fluid was evaluated using Franz diffusion cell. Membrane with 0.45 µ pores size (larger than nanocapsule size) does not have enough capability to control the permeation of active/nanocapsule. Therefore, this type of membrane is used for in vitro release test for liquid and semisolid products^37^. Cumulative % release of ferulic acid was plotted against time. Figure 6a indicates that active release rate from Gel A was fast in initial 3 h followed by slow release. Interestingly, active release rate in first 3 h was significantly slower from Gel B than Gel A. However, active release from Gel B continued after 3 h and cumulative % active release steadily increased. Overall, Gel B demonstrated controlled release of active (ferulic acid). In a study, Zheng et al. used same Carbopol polymer (Carbopol 934) at different level and showed substantial decrease in drug release with increasing polymer level in the gel.^50^. Generally, release/permeation rate of active decreases with increasing product viscosity/thickness caused by increasing polymer level. In our works, two different types of Carbopol polymers were selected based on the pH of the product. Thickening property of Carbopol at different pH depends on the type of Carbopol. Carbopol Ultrez 21 polymer has thickening property at relatively high pH after neutralization with base (i.e., base-swellable at pH > 4–5), while Carbopol Aqua CC polymer has thickening property at low pH after neutralization with acid (i.e., acid-swellable at pH < 4–4.5). Carbopol Ultrez 21 polymer is in powder form containing 100% polymer while Carbopol Aqua CC polymer is in liquid form containing 20% polymer. Therefore, actual polymer level was basically 2% when 10% (w/w) Carbopol Aqua CC was used. This higher level of Carbopol Aqua CC polymer was used to bring the thickness/viscosity of the gel to the acceptable level. Carbopol Aqua CC generates relatively low viscosity compared to other Carbopol polymers, such as Carbopol Ultrez 21. Carbopol Ultrez 21 was used at lower level than actual polymer level in Carbopol Aqua CC (i.e., 0.4% vs. 2%) as use of 2% Carbopol Ultrez 21 will make the gel very thick/viscous. The viscosity analysis of two gels showed that the viscosity of Gel A was significantly higher than Gel B, which means active release from Gel A should be slower than from Gel B if the release was controlled by product viscosity or thickness. On the other hand, aqueous solubility of ferulic acid increased with increasing pH. Therefore, part of ferulic acid is likely to exist as solution form (i.e., un-encapsulated) in Gel A (pH 5.43), which might have led faster active release compared to Gel B (pH 3.41). Hence, higher pH of Gel A than Gel B should be the major reason for faster active release from Gel A, although viscosity of the gels might have also played a role on the release profiles. Nevertheless, cumulative release after 24 h was not significantly different between Gel A and Gel B.

Active release from the gel formulations containing 0.5% (Gel B) and 0.25% (Gel C) nanoencapsulated ferulic acid were also compared. This experiment was conducted to find out whether % of active present in the gel has any impact on the release rate. No significant difference in cumulative % active release was noticed between these two gel formulations (Fig. 6b). This result indicates that active concentration did not have influence on cumulative % active release although cumulative amount of active release was significantly lower from gel containing 0.25% (w/w) ferulic acid than 0.5% (w/w) ferulic acid.

### Active permeation

**Figure 7:**
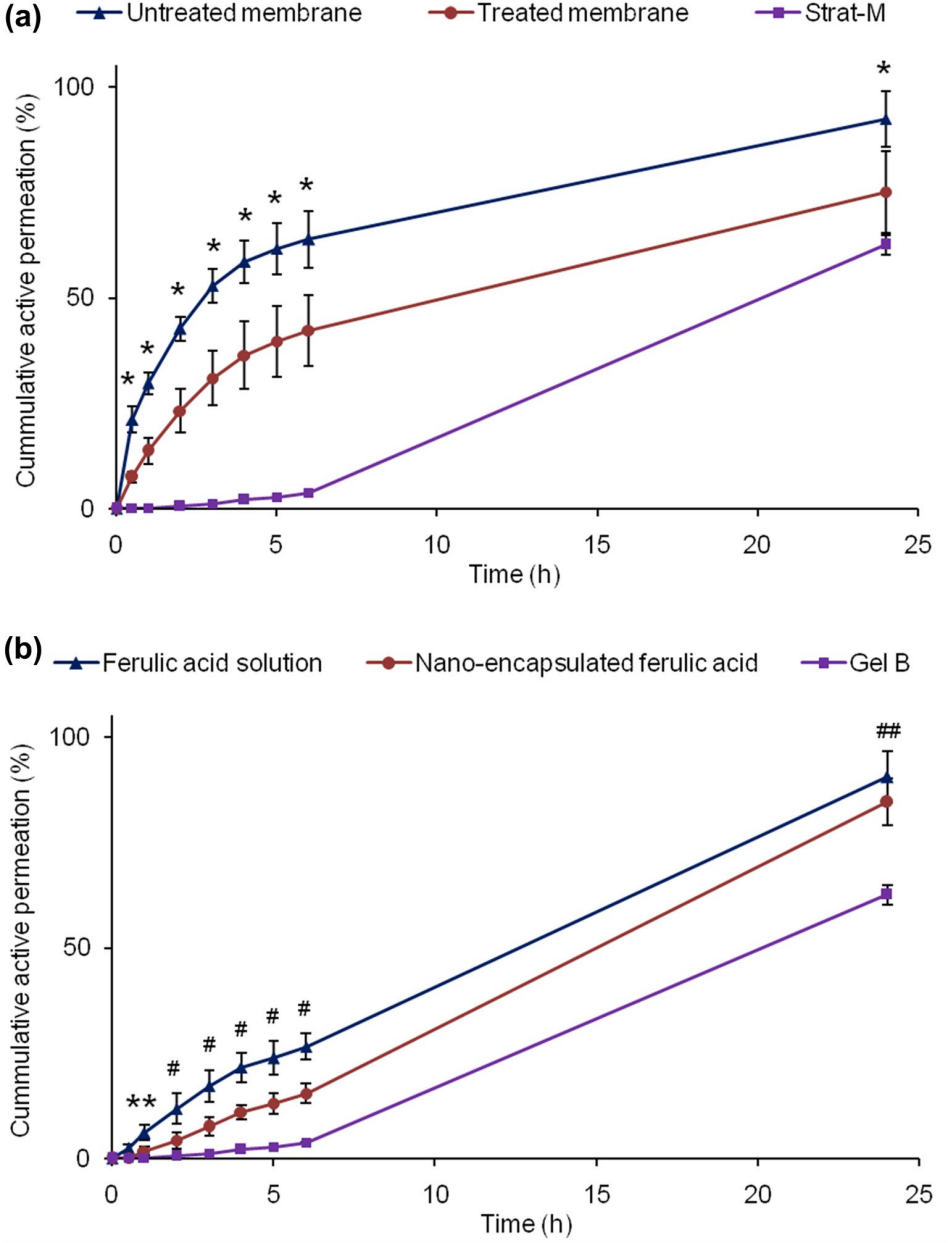
Cumulative active permeation (**a**) through treated and untreated polysulfone membranes, and Strat-M membrane (skin mimic) from Gel B, and (**b**) through Strat-M membrane from ferulic acid aqueous solution, nanoencapsulated ferulic acid dispersion, and Gel B. Data represent mean ± SD (n = 3). Significant difference (*p* < 0.05): *Untreated membrane versus treated membrane, treated membrane versus Strat-M, and untreated membrane versus Strat-M. ^#^Ferulic acid solution versus nanoencapsulated ferulic acid, nanoencapsulated ferulic acid versus Gel B, and ferulic acid solution versus Gel B. ^##^Gel B versus nanoencapsulated ferulic acid and ferulic acid solution. **Ferulic acid solution versus Gel B and nanoencapsulated ferulic.

In another study, the membrane was treated by soaking in 85% (w/w) isopropyl myristate (IPM) and 15% (w/w) ethoxylated aliphatic amine (Ethomeen O/12LC) for 12 h to simulate skin lipophilicity^44,51^. Any excess solution was removed by Kimwipe before its use on Franz diffusion cell. Gel B was used for this permeation study as it demonstrated controlled release and good physicochemical stability profiles (details in stability section). The result shows significantly lower active permeation rate through treated membrane than non-treated membrane (Fig. 7a). This is due to imposed lipophilicity on the membrane (closer to skin) which slowed down active permeation through the membrane. This result suggests that permeation of active will be controlled by structure and permeability of skin/membrane.

Furthermore, permeation of ferulic acid through Strat-M Membrane was also checked (Fig. 7a). Researchers have proven that Strat-M membrane can be used for active permeation study as alternative to skin^21^. Uchida et al. established similarity in chemicals’ permeation profile (e.g., permeability coefficient, permeation and diffusion parameters) between Strat-M and excised human skin and/or hairless rat skin by investigating permeation of thirteen chemical compounds with molecular weights of 152–289 and lipophilicities (log *K*_*O/W*_) of -0.9 to 3.5 via Franz diffusion cell studies^21^. Molecular weight and log *K*_*O/W*_ of ferulic acid are 194.18 and 1.51, respectively. Hence, permeation of ferulic acid through Strat-M is expected to provide similar permeation profile as human skin. Very low permeation was observed through Strat-M membrane till 6 h despite relatively higher permeation was observed through polysulfone membrane (i.e., high amount of ferulic acid was available at membrane surface). Other researchers also reported that active was not detected in systemic circulation following topical application of nanoparticles^45^. This result confirmed that ferulic acid permeation from the hydrogel formulation was controlled by the Strat-M structure which simulates skin structure from permeation point of view. Relatively high permeation through Strat-M membrane at 24 h indicates that active may slowly diffuse through different skin layers and reach to dermis. Penetration of active to deep skin layer (e.g., dermis and inner epidermis) is required for long lasting skin care benefits (e.g., anti-aging, anti-pigmentation). However, minimum or no active should arrive to systemic circulation to avoid undesirable effects. This permeation result showed sustained permeation of ferulic acid from hydrogel formulation, which is desirable for skin care and cosmetic products. Nevertheless, ferulic acid that does not penetrate into skin remains on skin surface, which may provide photo-protection benefit as ferulic acid can absorb UV light and act as sunscreen (reduce/prevent photo-ageing).

When permeation profiles of aqueous solution, nanodispersion and Gel B were compared, ferulic acid permeation was fastest from the aqueous solution followed by the nanodispersion (Fig. 7b). Ferulic acid permeation was slowest from Gel B. The results indicate that soluble ferulic acid at high pH can penetrate Strat-M membrane faster than nanodispersion. The release study also demonstrated higher active release from formulation with higher pH (Fig. 6a). Most importantly, slower permeation from Gel B than nanodispersion indicates that Carbopol was able to reduce/control ferulic acid permeation. Absence of any thickener (i.e., polymer) in the nanodispersion did not slow down the movement of nanocapsules and especially micelles, which might have led to faster membrane permeation from the nanodispersion than its gel form. Other researchers also observed substantial decrease in drug release with increasing Carbopol polymer (Carbopol 934) level in the gel^50^. This suggests that active permeation can be controlled by varying polymer level in the formulation.

## Stability

### Appearance

**Figure 8:**
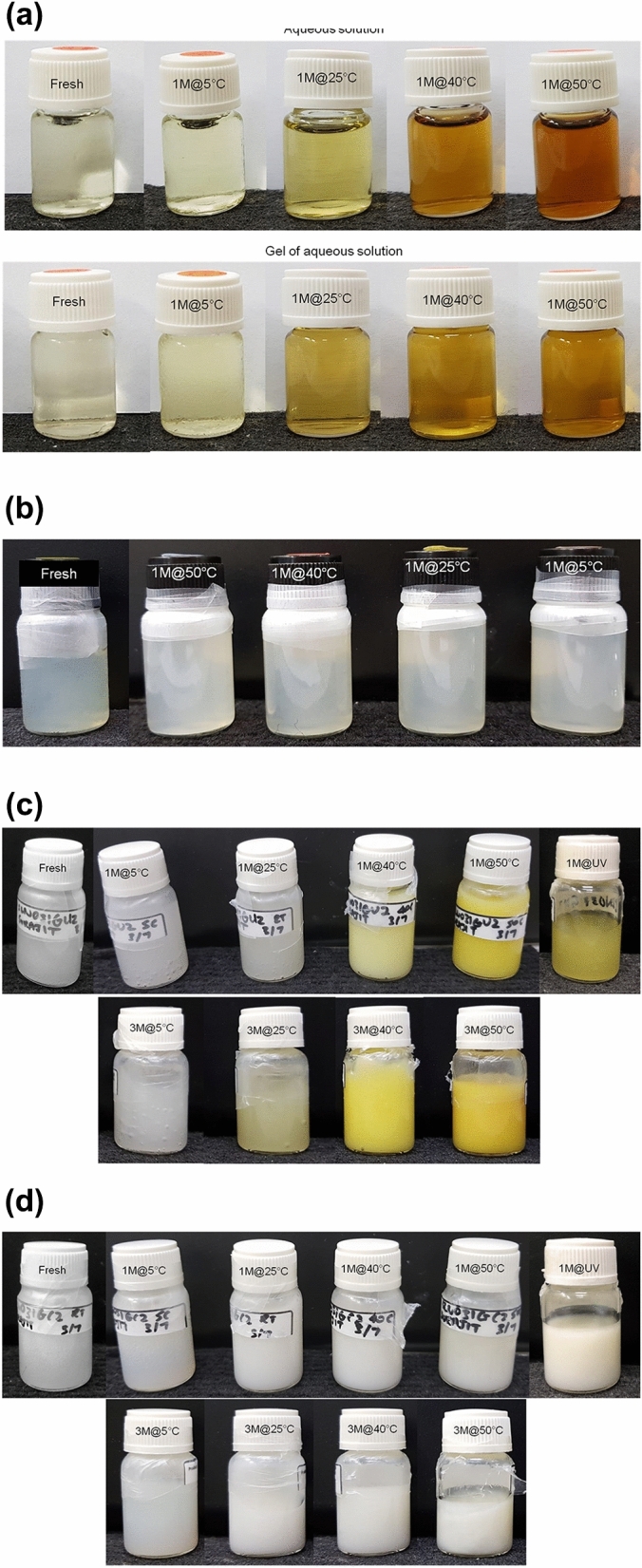
Appearance of (**a**) aqueous solution and gel of aqueous solution, (**b**) nanoencapsulated ferulic acid dispersion, (**c**) Gel A, and (**d**) Gel B stored at different storage conditions (5 °C, 25 °C, 40 °C/75% RH, 50 °C, and UV light) for one month (1 M) and three months (3 M). Fresh indicates fresh formulation that was prepared on the day of evaluation.

Figure 8a represents the appearance of ferulic acid aqueous solution and its gel formulation as fresh formulations and after their storage at 5 °C, 25 °C, 40 °C/75% RH, and 50 °C for one month. The images clearly demonstrate that the color of ferulic acid aqueous solution and its gel formulation significantly changed over time. Although insignificant color change was observed when stored at 5 °C, significant color change was observed when stored at 25 °C, 40 °C/75% RH, and 50 °C for one month. In fact, significant color change was evident even after one week storage at 25 °C, 40 °C/75% RH, and 50 °C. Intensity of color change increased with increasing storage temperature. The formulations turned yellowish at 25 °C, brownish at 40 °C/75% RH, and dark brownish at 50 °C after one month. This indicates serious stability and consumer acceptability issues of ferulic acid aqueous solution and its gel formulation. To prepare aqueous solution, ferulic acid was dissolved in alkaline solution (i.e., aqueous solution of sodium hydroxide) with pH > 8 as ferulic acid is either very slightly soluble or not soluble in water with neutral or acidic pH^17^. The xanthan gum-based reference gel was subsequently prepared by adding xanthan gum in the alkaline aqueous solution of ferulic acid. As discussed earlier, ferulic acid is highly unstable in alkaline environment, which led to significant degradation of ferulic acid causing dark color of aqueous solution and gel of aqueous solution. When nanoencapsulated ferulic acid dispersion was stored at 5 °C, 25 °C, 40 °C/75% RH, and 50 °C, insignificant color change was noticed after one month (Fig. 8b). This indicates that nanoencapsulation of ferulic acid was able to significantly improve color stability of ferulic acid compared to its alkaline aqueous solution.

Both Gel A and Gel B containing nanoencapsulated ferulic acid were also stored at 5 °C, 25 °C, 40 °C/75% RH, and 50 °C. Figure 8c shows the appearance of fresh Gel A and Gel A stored for one month at 5 °C, 25 °C, 40 °C/75% RH, and 50 °C. The image indicates that there was no visible color change of Gel A when stored at 5 °C for one month compare to fresh sample. Color slightly changed when stored at 25 °C for one month, while significantly changed when stored at 40 °C/75% RH and 50 °C for one month. Color of Gel A turned to light yellow and yellow when stored for one month at 40 °C/75% RH and 50 °C, respectively. However, the intensity of color shift was significantly lower than ferulic acid aqueous solution and its gel form (Fig. 8a). Appearance of Gel A stability samples was again checked after three months. There was no visible color change after three months when the gel was stored at 5 °C, whereas visible color change (yellowish) was observed when the gel was stored at 25 °C, 40 °C/75% RH, and 50 °C (Fig. 8c). The intensity of color change increased with increasing storage temperature. The intensity of color change was higher than one month stability samples of Gel A but still lower than the one month stability samples of ferulic acid aqueous solution and its gel form (Fig. 8a). This indicates that although color change issue during product shelf life was reduced in Gel A, color change was still obvious. Therefore, Gel A was unable to solve the consumer acceptability issue. The aqueous solution and its gel form turned brownish after stability period at accelerated temperatures (i.e., 40 °C and 50 °C), whereas Gel A turned to yellowish. Hence, there is a possibility that ferulic acid degraded differently and produced different degraded chemicals in different formulations. The pH of Gel A was higher than the p*K*_a_ of ferulic acid. Therefore, majority of ferulic acid molecules were de-protonated (i.e., ionized) and there is high chance that part of the ferulic acid gradually leaked outside the nanocapsules in Gel A due to higher aqueous solubility of ferulic acid above its p*K*_a_ value. This un-encapsulated ferulic acid most probably degraded and led color change of the gel. On the other hand, the pH of nanoencapsulated ferulic acid dispersion was < 4 (i.e., less than p*K*_a_ of ferulic acid), which might be the reason for its color stability compared to Gel A (Fig. 8b). In case of Gel B, no significant color change was noticed at all storage conditions (5 °C, 25 °C, 40 °C/75% RH, and 50 °C) after one month and three months (Fig. 8d). The pH of Gel B was lower than the p*K*_a_ of ferulic acid. Therefore, majority of ferulic acid was protonated (i.e., non-ionized) and aqueous solubility of ferulic acid was negligible at the pH of Gel B. Hence, most of the ferulic acid remained encapsulated in the nanocapsules, which prevented color change of the gel. This gel formulation was able to solve the consumer acceptability issue from product appearance point of view. Gel A and Gel B were also stored under UV light. Color of Gel A turned to yellowish after two weeks and turned to deep yellow after one month (Fig. 8c). On the other hand, color of Gel B did not significantly change even after one month when stored under UV light (Fig. 8d). This observation can be similarly explained by pH of the gels as discussed earlier. Ferulic acid might have partly leaked out in the external aqueous phase of Gel A, which led to direct exposure to UV light. On the other hand, ferulic acid remained encapsulated in Gel B and avoided direct exposure to UV light.

As observed, the color of the stability samples turned to light yellow to dark yellow and light brown to dark brown depending on the formulation type and storage condition. Such color changes might be due to degradation of ferulic acid into same degraded molecules at different level and/or different degraded molecules^5,8,13^. Carbopol polymers may also have partial influence in ferulic acid stability/degradation.

### pH

The pH of both Gel A and Gel B was measured on fresh sample as well as after one month and three months storage at 40 °C/75% RH (commonly used in the industries as accelerated stability condition). No significant change in pH was observed at any time point for both Gel A and Gel B (Supplementary Figure S3). For Gel A, initial pH of 5.43 ± 0.11 became 5.37 ± 0.05 and 5.41 ± 0.02 after one and three months, respectively. For Gel B, initial pH of 3.41 ± 0.07 became 3.31 ± 0.10 and 3.30 ± 0.03 after one and three months, respectively.

### Active content

**Figure 9:**
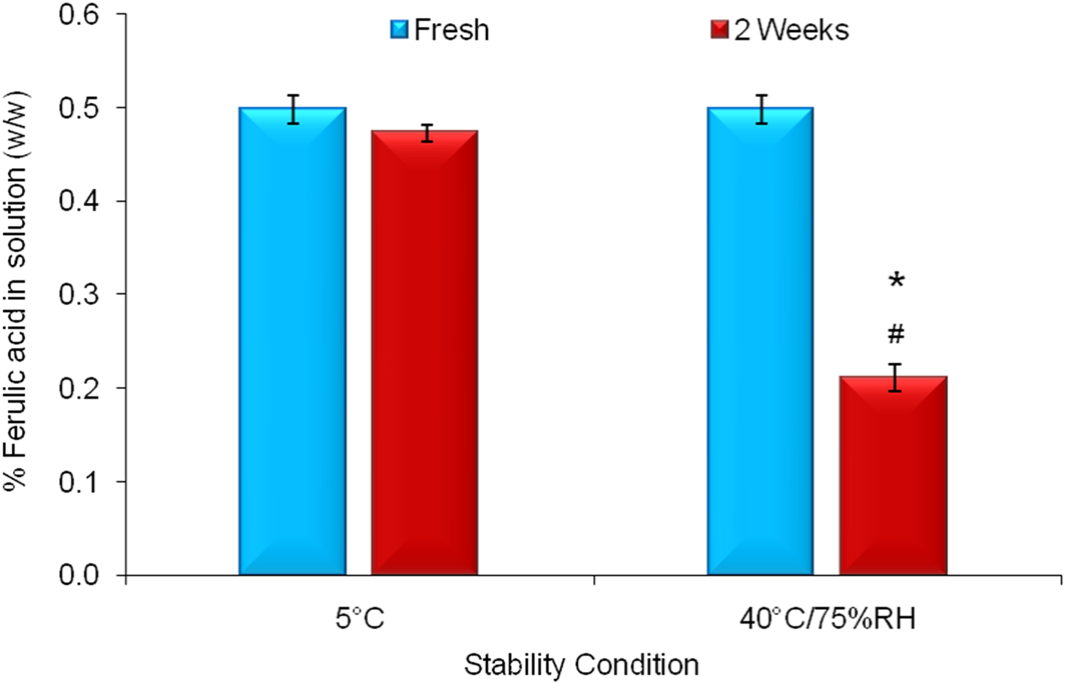
Stability profile of ferulic acid aqueous solution at 5 °C and 40 °C/75% RH. Data represent mean ± SD (n = 3). Significant difference (*p* < 0.05): *Fresh versus 2 weeks at 40 °C/75% RH. ^#^2 weeks at 40 °C/75% RH versus 2 weeks at 5 °C.

Active (ferulic acid) contents of ferulic acid aqueous solution stored at 5 °C and 40 °C/75% RH for two weeks were analyzed via HPLC and compared with the active content of fresh solution. The result indicates that the active content was > 95% for the sample stored at 5 °C, whereas active content was < 43% for the sample stored at 40 °C/75% RH (Fig. 9). As mentioned earlier, color of the solution also turns brownish when stored at 40 °C/75% RH. This proves that ferulic acid was not stable in this aqueous solution form.

**Figure 10:**
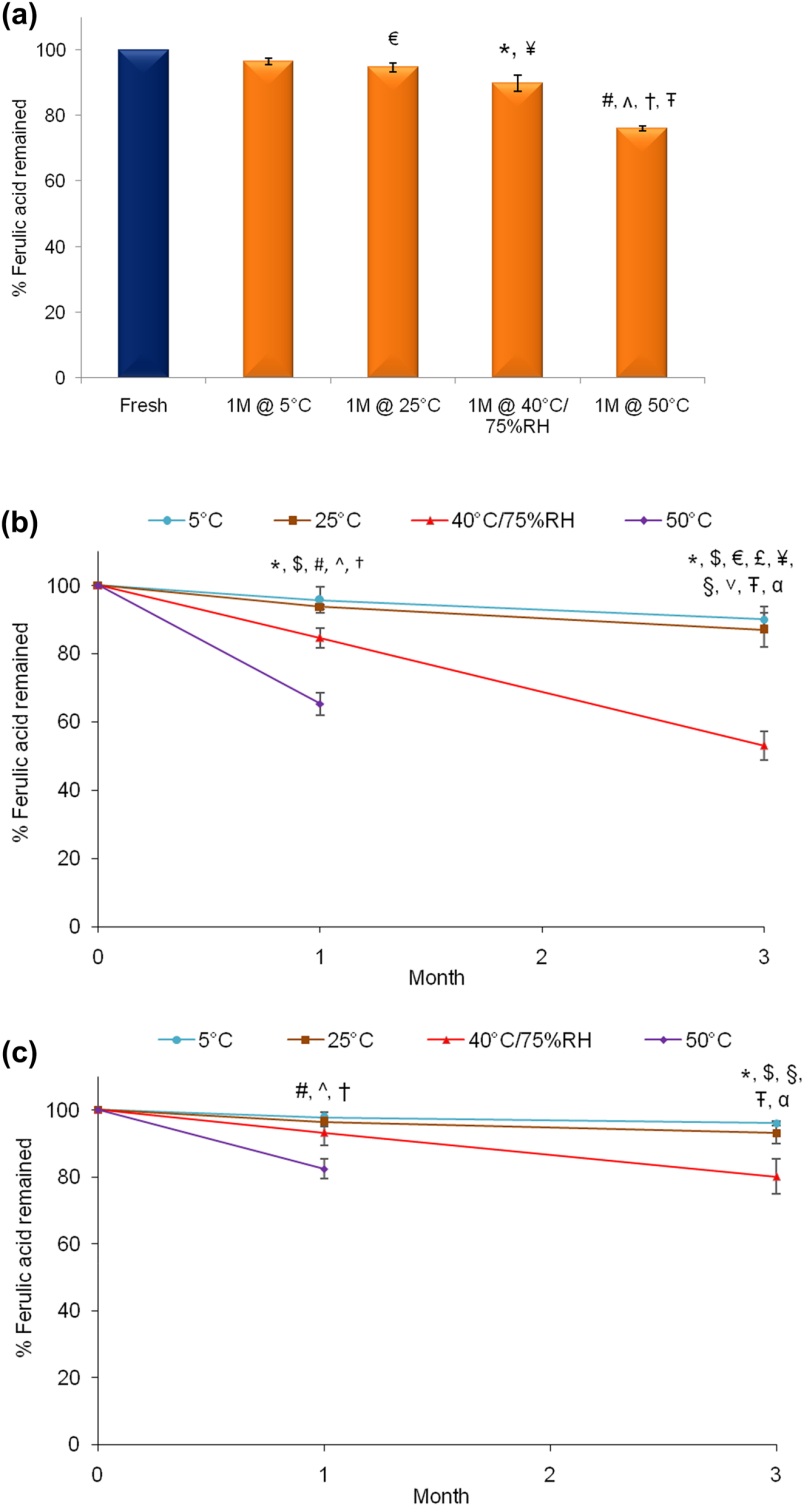
Stability profile of ferulic acid in (**a**) nanoencapsulated ferulic acid dispersion, (**b**) Gel A, and (**c**) Gel B stored at 5 °C, 25 °C, 40 °C/75% RH, and 50 °C. Data represent mean ± SD (n = 3). Significant difference (*p* < 0.05): ^#^5 °C versus 50 °C, *5 °C versus 40 °C, ^$^25 °C versus 40 °C, ^25 °C versus 50 °C, ^†^40 °C versus 50 °C, ^€^Fresh versus 1 M @ 25 °C, ^¥^ Fresh versus 1 M @ 40 °C, ^Ŧ^Fresh versus 1 M @ 50 °C, ^α^ Fresh versus 3 M @ 5 °C, ^£^Fresh versus 3 M @ 25 °C, ^§^Fresh versus 3 M @ 40 °C, and ^˅^1 M @ 40 °C versus 3 M @ 40 °C.

Stability of nanoencapsulated ferulic acid dispersion was evaluated till one month to investigate whether nanoencapsulation of ferulic acid can improve its chemical stability (Fig. 10a). Ferulic acid was reasonably stable in nanoencapsulated ferulic acid dispersion at 5 °C and 25 °C (> 96% and ~ 94%, respectively). The result showed significant improvement of ferulic acid stability following nanoencapsulation compared to aqueous solution (~ 90% after one month vs. ~ 42% after two weeks at 40 °C/75% RH, respectively). Nanoencapsulation was able to improve stability of ferulic acid even when was stored at 50 °C (> 76% after one month).

Figure 10b depicts the stability profile of ferulic acid in Gel A at different storage conditions. It shows that active content of Gel A stored at 5 °C and 25 °C slightly decreased after one month (~ 95% and ~ 93% remained, respectively) and further decreased after three months (~ 90% and ~ 87% remained, respectively). At 40 °C/75% RH, active content significantly decreased after one month (~ 84% remained) and further decreased after three months (< 53% remained). The degradation of ferulic acid in Gel A was fast when stored at 50 °C (~ 65% remained after one month). The degradation of ferulic acid increased with increasing storage temperature and duration. This observation is consistent with appearance (color) change (Fig. 8c). However, the data clearly shows that the stability of ferulic acid significantly improved in Gel A (contain nanoencapsulated ferulic acid) in compare to ferulic acid aqueous solution (Fig. 9). Unfortunately, stability improvement was below desirable level. Chemical stability of ferulic acid in Gel A was significantly lower than in nanoencapsulated ferulic acid dispersion, especially at 40 °C/75% RH and 50 °C (Fig. 10a). As discussed earlier, this might be due to higher pH of Gel A (pH ~ 5.4) than nanoencapsulated ferulic acid dispersion (pH < 4).

Similarly, Gel B was also stored at similar stability conditions and stability of ferulic acid was analyzed at each time points (Fig. 10c). Only small amount of active degradation was observed at 5 °C and 25 °C after one month (> 97% and > 96% remained, respectively). Active contents at 5 °C and 25 °C after three months were > 96% and > 93%, respectively. Active content decreased over time at 40 °C/75% RH (> 93% remained after one month and > 80% remained after three months). Active content at 50 °C was > 82% after one month. The stability of ferulic acid in Gel B was substantially better than nanoencapsulated ferulic acid dispersion at 40 °C/75% RH and 50 °C (Fig. 10a). Therefore, gel formulation at low pH (even lower than nanoencapsulated ferulic acid dispersion) was able to improve the stability of nanoencapsulated ferulic acid. Other researchers reported less drug leakage/release from liposomes during stability periods when formulated with increased level of Carbopol gel (although the gel was alkaline contrary to acidic Gel B)^52^. Overall, stability of ferulic acid in Gel B was significantly better than Gel A at all storage conditions. As discussed earlier, part of ferulic acid probably leached out from the nanocapsules in Gel A as pH of Gel A was higher than ferulic acid’s p*K*_a_ value (aqueous solubility of ferulic acid increased with increasing pH). This un-encapsulated soluble ferulic acid was prone to degradation over time (especially at high temperature). On the other hand, most of the ferulic acid might have remained encapsulated in Gel B as pH of Gel B was lower than ferulic acid’s p*K*_a_ value (ferulic acid is insoluble in water at low pH). This might be the reason for better stability of ferulic acid in Gel B than Gel A. Type and concentration of Carbopol in the gel might also have partial effect on stability of ferulic acid as other researchers observed that Carbopol reduced drug leakage from liposomal formuation^52^.

## Conclusion

Nanoencapsulation of ferulic acid was performed and physicochemical characterizations indicated successful encapsulation of ferulic acid. Topical gel formulations containing nanoencapsulated ferulic acid were prepared. The level of ferulic acid in the gel formulations was similar to marketed products. This study proved that nanoencapsulation of ferulic acid significantly improved the stability of ferulic acid in the formulation. Furthermore, nanoencapsulated ferulic acid containing gel formulation with low pH (lower than p*Ka* of ferulic acid) prevented color change of the gel formulation as well as significantly improved the stability of ferulic acid. This proves that pH of the formulation containing nanoencapsulated ferulic acid is a crucial factor. The release studies indicated that the release rate of ferulic acid was influenced by the pH of the gel formulations. Most importantly, the cumulative % of ferulic acid permeation was very low till 6 h when skin mimic (Strat-M) was used. This proves that ferulic acid penetration will be restricted by the membrane/skin structure despite its sufficient availability on skin/membrane surface. Relatively high permeation of ferulic acid at 24 h suggests that active continued to slowly penetrate through the membrane layers. For chronic skin care efficacy (e.g., anti-aging, skin lightening, etc.) in long run, active penetration to deep skin layers (inner epidermis and dermis) is important while minimum or no active should reach to systemic circulation to avoid undesirable side effects. Furthermore, it has been well established that the skin care product with low pH is beneficial for skin. In summary, combined approach of nanoencapsulation and low pH was able to stabilize ferulic acid in the hydrogel formulation, while ferulic acid may penetrate into deep skin layers but avoid systemic circulation. The developed topical hydrogel formulation shows huge potential for commercialization.

## Supplementary information

Supplementary file1 (DOCX 457 kb)

## Data Availability

The data sets and analysis of current study are available from the corresponding author upon reasonable request.
