# Supplementary material for: Stabilization of ferulic acid in topical gel formulation via nanoencapsulation and pH optimization
Source: Sci Rep. 2020 Jul 23;10:12288. doi: 10.1038/s41598-020-68732-6 (PMC7378829; doi:10.1038/s41598-020-68732-6)
Supplement: Supplementary file 1 — Supplementary file1 (DOCX 457 kb) [file 41598_2020_68732_MOESM1_ESM.docx]

**Stabilization of ferulic acid in topical gel formulation via nanoencapsulation and pH optimization**

Surajit Das^†^, Annie Wong

Institute of Chemical and Engineering Sciences, A*STAR (Agency for Science, Technology and Research), 1 Pesek Road, Jurong Island, Singapore 627833

**Corresponding author**

Institute of Chemical and Engineering Sciences, A*STAR (Agency for Science, Technology and Research), 1 Pesek Road, Jurong Island, Singapore 627833

^†^ Tel: (65) 6796 3719, Fax: (65) 6316 6183, E-mail: surajit_das@ices.a-star.edu.sg; surajitdas1982@yahoo.com

a)

b)

**Supplementary Figure S1:** Particle size and polydispersity index (PdI) of the nanocapsules a) without dilution, and b) with 10-times dilution. Data represent mean ± SD (n = 3).

* Significant difference in particle size (p <0.05).

** Significant difference in PdI (p <0.05).

a)


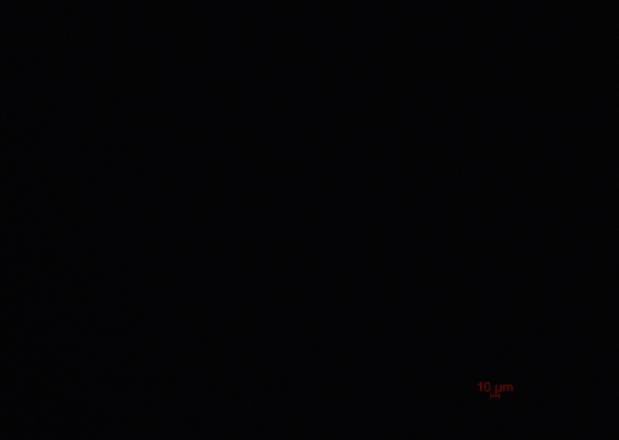


b)


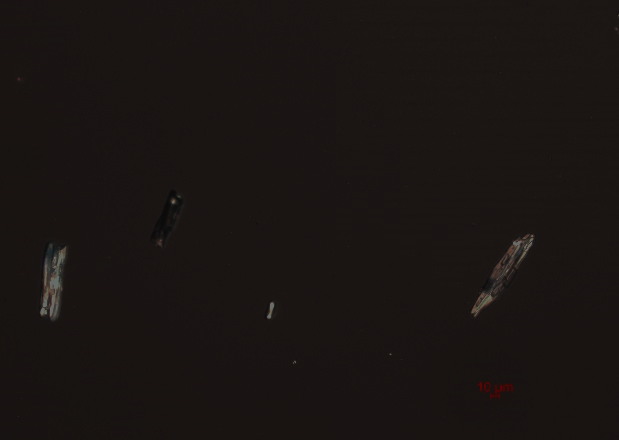


**Supplementary Figure S2:** Cross polarized microscopic image of a) Gel B containing nanoencapsulated ferulic acid that was stored at 5 °C for three months and b) gel containing ferulic acid powder.


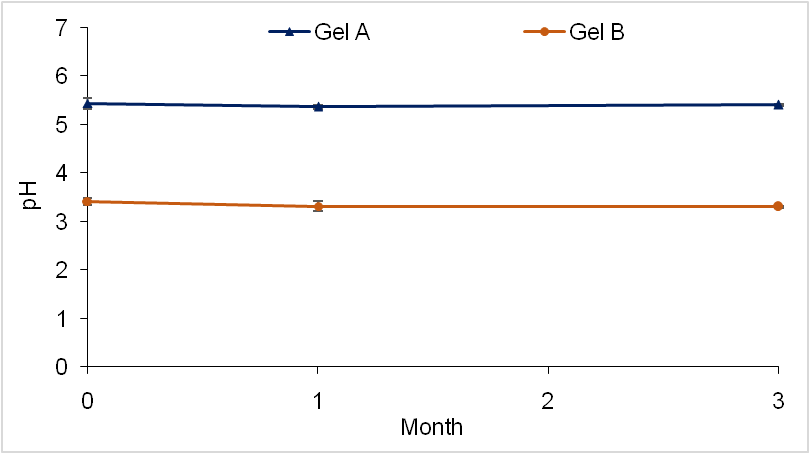


**Supplementary Figure S3:** The pH profile of Gel A and Gel B stored at 40 °C/75 % RH. Data represent mean ± SD (n = 3).
